# War and Fever

**Published:** 1899-12-02

**Authors:** 


### WAR AND FEVER.

For many reasons the subject of typhoid fever is at the present moment one of great interest and importance. Here at home its prevalence in London has for several weeks rapidly increased, and although this outbreak seems now to be as rapidly passing away the question how far it may or may not be due to the source of our water supply is engaging considerable attention; while in South Africa one of the great dangers, perhaps the greatest danger, to which our army is exposed is this ever-present foe, typhoid fever. Surgeon-General Jameson, Director-General of the Army Medical Service, speaking at a dinner a week or two ago, said: "In this war there are two powerful enemies to contend with—the Boers and enteric fever"; and, to show to his hearers how serious a matter this enteric or typhoid fever is to armies in the field, he went on to state that in a letter from the Director-General of the United States Army Medical Department which he had just received it was mentioned that in their late war in Cuba enteric fever was much more deadly to their troops than gunshot wounds. The disastrous effects produced by typhoid fever on the troops engaged in Cuba are now thoroughly recognised. Speaking on this subject recently, Dr. Hermann Biggs, of New York, said: "In the face of all the modern progress of the knowledge of the infectious diseases, and the means for their prevention, and in spite of the extraordinary advances of sanitary science in all its phases, hundreds and thousands of our soldiers sickened and died of this disease, and many returning home while ill, or during convalescence, carried with them and spread broadcast typhoid infection in towns, villages, and rural districts throughout the United States."

Typhoid fever has always been one of the scourges of South Africa. Even during last year, in time of peace, the admission rate among our troops in Natal was $5\frac{1}{2}$ per cent., and that among healthy, picked men, and we know only too well that all movement of troops and all aggregation of masses of men, especially in countries where the water supply oscillates between scarcity and floods, tends infinitely to increase the tendency of this disease to take upon itself an epidemic form. Already, even with the very limited amount of telegraphic communication which is permitted, we hear of deaths from typhoid among our men, and we hear of just that sort of difficulty about water which is apt to end in widespread prevalence of this form of fever. There can be no doubt, then, that there is much cause for anxiety.

Of course everything is being done. Filters of approved pattern have been supplied in large numbers. A very considerable proportion of the troops who have gone out have been inoculated—a proceeding which, according to the latest reports, seems to give a considerable amount of protection, at any rate, for a time—and we may be quite sure that our army medical officers, who are to be regarded as sanitary officers also, are thoroughly imbued with the necessity of doing everything that is possible to keep this disease at bay. But, however carefully our camps may be kept and their water supply safeguarded, there are times when troops on active service, in the frightful thirst engendered by marching under such a sun as glares down upon South Africa, cannot be restrained from partaking of almost the first water that comes to hand, and, moreover, the fact is becoming every year more clear that, although in well-kept barracks water is practically the only danger, so that typhoid can be abolished from them by the use of proper filters, in the rough and tumble of camp life there are many other sources of infection. The flies, the dust, the lack of appliances for ensuring cleanliness, all offer endless opportunities for the conveyance of infected matter to the food or drink of those living in such conditions, and in these things lie dangers which it is impossible wholly to overcome. When we recall how prevalent typhoid fever is among our soldiers in India, notwithstanding all the care that is lavished upon them, and how even that model campaign, the advance to Khartoum, under Lord Kitchener, ended with a smart outbreak of this disease; and when we recall, again, the fact that typhoid fever is, as it has been well called, the scourge of South Africa—a disease which even in peace falls upon our troops there far more severely than upon those in other parts—we cannot avoid a feeling of anxiety. At present this country is in the midst of a great access of patriotism, and our brave fellows out in South Africa are, with unfailing gallantry, facing wounds and sudden death for glory and their country. But wounds are not the only penalties of warfare, and those who know most concerning fever pray the most earnestly that this war may soon be over.

